# Enhanced accumulation of phenolics in pea (*Pisum sativum* L.) seeds upon foliar application of selenate or zinc oxide

**DOI:** 10.3389/fnut.2023.1083253

**Published:** 2023-03-30

**Authors:** Maksymilian Malka, Gijs Du Laing, Gabriela Kurešová, Alžbeta Hegedüsová, Torsten Bohn

**Affiliations:** ^1^Laboratory of Analytical Chemistry and Applied Ecochemistry, Department of Green Chemistry and Technology, Faculty of Bioscience Engineering, Ghent University, Ghent, Belgium; ^2^Department of Plant Protection, Crop Research Institute, Prague, Czechia; ^3^Institute of Horticulture, Faculty of Horticulture and Landscape Engineering, Slovak University of Agriculture in Nitra, Nitra, Slovakia; ^4^Nutrition and Health Research Group, Department of Precision Health, Luxembourg Institute of Health, Strassen, Luxembourg

**Keywords:** oxidative stress, micronutrients, trace elements, polyphenols, legume biofortification, food security, mineral deficiency, foliar Se/Zn application

## Abstract

**Background:**

Selenium (Se) and zinc (Zn) are essential antioxidant enzyme cofactors. Foliar Se/Zn application is a highly effective method of plant biofortification. However, little is known about the effect of such applications on the concentration of trace elements and phytochemicals with pro-oxidant or antioxidant activity in pea (*Pisum sativum* L.).

**Methods:**

A 2-year pot experiment (2014/2015) was conducted to examine the response of two pea varieties (Ambassador and Premium) to foliar-administered sodium selenate (0/50/100 g Se/ha) and zinc oxide (0/375/750 g Zn/ha) at the flowering stage. Concentrations of selected trace elements (Fe, Cu, and Mn), total phenolic content (TPC), total flavonoid content (TFC), and total antioxidant activity (ABTS, FRAP) of seeds were determined.

**Results and conclusions:**

Se/Zn treatments did not improve the concentration of trace elements, while they generally enhanced TPC. Among examined treatments, the highest TPC was found in Ambassador (from 2014) treated with 100 g Se/ha and 750 g Zn/ha (2,926 and 3,221 mg/100 g DW, respectively) vs. the control (1,737 mg/100 g DW). In addition, 50 g of Se/ha increased TFC vs. the control (261 vs. 151 mg/100 g DW) in Premium (from 2014), 750 g of Zn/ha increased ABTS vs. the control (59.5 vs. 25.2 mg/100 g DW) in Ambassador (from 2015), and 50 g of Se/ha increased FRAP vs. the control (26.6 vs. 18.0 mmol/100 g DW) in Ambassador (from 2015). In linear multivariable regression models, Zn, Mn, Cu, and TPC best explained ABTS (*R* = 0.577), while Se, Cu, and TPC best explained the FRAP findings (*R* = 0.696). This study highlights the potential of foliar biofortification with trace elements for producing pea/pea products rich in bioactive plant metabolites beneficial for human health.

## 1. Introduction

Phenolic compounds or polyphenols are organic (poly)hydroxylated compounds, which are widely abundant bioactive secondary plant metabolites (1). It is estimated that >8,000 phenolic compounds have been identified in plants (2, 3). These are recognized for their many different functions in plants, including their importance for pigmentation and sensory characteristics, growth and reproduction, and resistance against biotic and abiotic stress conditions (4–7). The concentration of phenolic compounds in plant sources depends on numerous factors, such as the biological background (genotype, organ, and ontogeny), cultivation techniques, growing conditions, ripening process, processing, storage conditions (8, 9), as well as extraction methods (10).

The current literature shows that the intake of phenolic compounds originating from various natural sources is related to antioxidant and anti-inflammatory activities, and may protect against a number of chronic diseases, such as cancer, type 2 diabetes, and other cardiometabolic complications, as well as against osteoporosis, pancreatitis, and gastrointestinal problems (11–13). A large body of research shows that the health benefits of phenolic compounds are linked to their antioxidant properties. These are regarded for their free radical scavenging activity, stabilization of divalent cations, and alteration of antioxidant enzymes, e.g., superoxide dismutase (SOD), catalase (CAT), and glutathione peroxidase (GPx), in part *via* their interactions with transcription factors, such as Nrf-2 (14–16).

Selenium (Se) is an essential trace element for humans. It is an integral component of Se-dependent antioxidant enzymes termed selenoproteins, such as GPx and thioredoxin reductases (TrxR), carrying out a variety of diverse functions. Selenoproteins are involved in antioxidant defense (e.g., within GPx), immune function (e.g., for T-cell proliferation), and thyroid hormone metabolism (e.g., for iodothyronine deiodinase), among others (17–19).

Zinc (Zn) is the second most abundant trace element [after iron (Fe)] and is essential for all living organisms (20). In humans, >300 enzymes and 2,000 transcription factors are known to require Zn. Zinc acts as a cofactor for various antioxidant enzymes, such as SOD and CAT (21). It plays important roles in cellular homeostasis, the immune system, brain and liver function, and metabolic processes, such as carbohydrate, lipid, protein, and nucleic acid synthesis or degradation (21–24).

A low intake of Se (as selenoproteins) and Zn or their low circulating concentrations have been linked with an increased risk of mortality and various chronic illnesses, such as several types of cancer, neurodegenerative diseases, type 2 diabetes, metabolic syndrome, and other cardiometabolic complications (17, 19, 22). Low Se and Zn statuses have also been associated with infectious diseases, including the recent COVID-19 (25, 26).

Pea (*Pisum sativum* L.) is an important legume widely grown and consumed throughout the world. The crop is used for animal and human nutrition. Pulses (including peas) are insufficiently represented in agricultural research, although they have been recognized for their contributions to sustainable agriculture and environment, biodiversity, public health, and food security (27–29). Peas are an important, inexpensive, and readily available source of proteins (30), carbohydrates, dietary fiber, starch, vitamins, minerals, and phytochemicals, which may be beneficial for human health and wellbeing (1, 31, 32). Phenolic compounds are the best-characterized phytochemicals in peas (31). In peas, at least 115 various polyphenols have been identified so far, constituting mostly glycosylated flavonols, together with biosynthetically related compounds, such as anthocyanins, flavones, flavanols, and flavanones (33). Clinical studies have shown that the consumption of peas and pea constituents may improve metabolic, cardiovascular, and gastrointestinal conditions in humans (31, 34). However, research dedicated to the possible health effects of pea phytochemicals (including phenolic compounds) is scarce. For example, *in vitro* anti-cancer activities of phenolic compounds from seed coats of pea were reported by Stanisavljević et al. (35). Another study emphasized the anti-cancer potential of flavonoids from pea peels (36).

Foliar spraying is a very effective method of plant biofortification for Se and Zn (37, 38). However, little information is available regarding the potential effects of trace elements on phytochemical antioxidants in pea that may contribute to health benefits in humans. Owing to their implications in many metabolic pathways, increased Se and Zn bioavailability in the plant could impact the accumulation of trace elements and bioactive secondary metabolites in plant tissues.

The aim of this 2-year pot experiment was to examine the response of pea varieties (Ambassador and Premium) to foliar-applied sodium selenate and zinc oxide at the flowering stage. The experiment consisted of five treatments, including one control (no fertilization) and two levels of foliar applications of both Se and Zn. Accumulation of selected trace elements and phytochemicals in seeds, related to anti-inflammatory and antioxidant processes, together with total antioxidant activity and their respective correlations, were determined.

## 2. Materials and methods

### 2.1. Chemicals

Zinkuran SC was acquired from Arysta LifeScience Slovakia s.r.o. (Nové Zámky, Slovakia). Sodium selenate was purchased from Alfa Aesar (Karlsruhe, Germany). Nitric acid (HNO_3_, for trace element analysis) was obtained from LGC Standards (Molsheim, France), and hydrogen peroxide (H_2_O_2_) 30% (Suprapur) was obtained from Merck/VWR (Leuven, Belgium). Methanol was purchased from Biosolve (Valkenswaard, the Netherlands). Acetone, sodium carbonate (Na_2_CO_3_), and sodium hydroxide (NaOH) were acquired from Merck (Darmstadt, Germany). Ascorbic acid (vitamin C), (+)-catechin, gallic acid, Folin–Ciocalteu's phenol reagent, 2,4,6-tri(2-pyridyl)-s-triazine (TPTZ), 2,2′-azino-bis(3-ethylbenzothiazoline-6-sulfonic acid) (ABTS) diammonium salt, 2–2′-azobis (2-methylpropionamidine) dichloride (AAPH), aluminum chloride (AlCl_3_), and iron(II) sulfate were obtained from Sigma–Aldrich (St. Louis, MO, USA). Hydrogen chloride (HCl) and sodium nitrite (NaNO_2_) were obtained from VWR (Leuven, Belgium). Iron(III) chloride 6-hydrate was purchased from Merck (Darmstadt, Germany).

### 2.2. Experimental design and sample preparation

A 2-year outdoor pot experiment was conducted between March and June for two growing seasons (2014 and 2015) in the Botanical Garden of the Slovak University of Agriculture, Nitra, Slovakia (48.305 N, 18.096 E). The experiment was designed with four replicates per treatment, two pea varieties, 2 years, and five different treatments (employing a total of 80 pots), so that three main factors were studied. The chemical and physical properties of the tested soil (including employed methods), and climate conditions are provided in our previous study (39). Two dark-seeded and high-yielding pea varieties were used, namely Ambassador (late variety, restored hybrid) and Premium (early variety, open pollinated). Seeds were obtained from a local farmer. Ten L plastic square pots were filled with soil and protected by a wire mesh against birds. In total, 30 seeds/pot were sown at a depth of 5 cm in two rows in mid-March. Selenium as sodium selenate and Zn as Zinkuran SC (30% ZnO + 6% chelate) were applied in the experiment. No additional fertilization was employed according to the recommendations of pea farmers and earlier studies (40). The five treatments evaluated were control (no fertilization), 50 g of Se/ha (Se1), 100 g of Se/ha (Se2), 375 g of Zn/ha (Zn1), and 750 g of Zn/ha (Zn2). The solutions used contained 0.1 and 0.2 g/L of Se and 0.75 and 1.5 g/L of Zn. Foliar application with Se and Zn was carried out on a single occasion during the time of plant flowering and the absence of rain. The timing of foliar applications was chosen according to the previous investigation (40). A plastic trigger spray bottle was used for the manual application of fertilizers. Irrigation and phytosanitary control were applied regularly, following daily visual controls. No toxic effects of Se and Zn treatments on plants were reported during the experiment. No incidences of pests and diseases were observed. Freshly manually harvested seeds at physiological maturity were immediately lyophilized and homogenized by grinding, and the concentration of Fe, Cu, and Mn, total phenolic content (TPC), total flavonoid content (TFC), and antioxidant activities (ABTS and FRAP) were examined.

### 2.3. Concentration of trace elements

Sample aliquots (0.2 g) were mixed with 3.5 ml of each HNO_3_ (65%) and H_2_O_2_ (30%). Then, microwave digestion was carried out using a MARS 6 system (CEM, Orsay Cedex, France, 1,200 W, 10 min at 55°C, 10 min at 75°C, and 45 min at 120°C). Concentrations of Fe, Cu, and Mn [mg/kg dry weight (DW)] were determined through the water-diluted digests *via* an ICP-OES (Varian Vista MPX, Palo Alto, CA, USA). For quantification, external calibration was applied. Regular measurement of calibrated blanks was carried out to monitor accuracy and precision during sample runs. For this purpose, certified reference materials (rice flour NIST1568a, sea lettuce BCR279, and spinach leaves SRM 1570a) were employed.

### 2.4. Extraction of phenolic compounds

The method published by Bouayed et al. (41) was employed to extract phenolic compounds (including flavonoids and ABTS determination) from dry material. In brief, 0.5–1 g of freeze-dried pea material was weighted in a 15 ml screw-cap falcon tube, to which 7.5 ml of 80% (v/v) methanol was added. The tubes were sonicated for 5 min and centrifuged (4,000 g, 5 min, RT, Heraeus Multifuge X3, Thermo Scientific, Leuven). Supernatants were removed and placed into a new 15 ml screw-cap falcon tube. Residues from the first tube were re-extracted once with 3 ml of 80% methanol, mixed, sonicated (5 min), and then centrifuged (4,000 g, 5 min). Both supernatants were combined, and this step was repeated once. The final extract was evaporated under nitrogen using a Turbovap blower (Caliper Life Sciences, Teralfene, Belgium) until ~2 ml remained (if needed, distilled water was added to a certain volume). Methanol (100%) was used to adjust to a final volume (4 ml). Extracts were overlayered with argon and stored at −80°C until further analyses.

### 2.5. Extraction procedure for FRAP determination

The method described by Kaulmann et al. (42) was used. In brief, freeze-dried pea material (100 mg) was weighed into 15 ml plastic tubes, to which 2 ml of acetone was added, vortexed (1 min), sonicated (5 min), put for 5 min on ice, and centrifuged (2 min, 2,500 g, 4°C). The supernatant was removed and placed in a 50 ml plastic tube. The residue was re-extracted (four times with 2 ml of acetone). The combined extracts were mixed briefly, and the volume of acetone was noted down. An aliquot of this volume was filtered through a 0.45 μm nylon filter. Unused samples were stored under argon at −80°C.

### 2.6. Determination of total phenolics

Total phenolic content was measured spectrophotometrically, using Folin–Ciocalteu's phenol reagent and employing gallic acid as standard (41). In short, 200 μl of appropriately diluted extract or standard and 200 μl of Folin–Ciocalteu's phenol reagent were added to a 10 ml centrifuge tube containing 1.8 ml of water and vortexed. After 5 min of incubation, 2 ml of 7% (w/v) Na_2_CO_3_ was added, and the solution was immediately diluted to 5 ml with water and mixed well. The mixture was incubated (90 min, RT), and the absorbance was measured at 750 nm against the blank. Quantification was based on external calibration curves (gallic acid, *n* = 6 concentrations between 0 and 100 mg/L) to determine the total phenolic content expressed as gallic acid equivalents (GAE) per 100 g DW.

### 2.7. Determination of total flavonoids

The total flavonoid content was assessed by spectrophotometry with catechin as a standard (41). In short, 200 μl of appropriately diluted extract or standard was added to 15 ml centrifuge vials containing 800 μl of water. Next, 60 μl of 5% (w/v) NaNO_2_ was added, and the vial was mixed. After 5 min, 60 μl of 10% AlCl_3_ was added. After 6 min, 400 μl of NaOH (1 M) was added, and the mixture was diluted to 2 ml with water. The absorbance was measured at 510 nm vs. the blank. Quantification was based on external calibration curves (catechin, *n* = 6 concentrations between 0 and 100 mg/L), and the total flavonoid content was expressed as catechin equivalents (CE) per 100 g DW.

### 2.8. Estimation of antioxidant capacity (ABTS)

The ABTS-radical scavenging capacity assay was examined using the vitamin C equivalent antioxidant capacity (VCEAC) test (41). In brief, 2.5 mM ABTS was mixed with 1 mM AAPH in phosphate-buffered saline (pH 7.4). The mixture was heated (water bath, at 68°C, ca. 15 min) to receive a blue–green ABTS radical solution. The absorbance of this solution was adjusted to 0.650 ± 0.020 with further phosphate-buffered saline (pH 7.4). Then, 40 μl of diluted extract or standard was mixed with 1,960 μl of ABTS radical solution. This was incubated in the dark at 37°C for 10 min. In the following, the absorbance was read at 734 nm vs. the blank. Quantification was performed by external calibration curves (vitamin C, *n* = 5 concentrations between 0 and 0.2 mg/L) to determine the antioxidant capacity expressed as vitamin C equivalents (VCE) per 100 g DW.

### 2.9. Estimation of antioxidant capacity (FRAP)

The ferric-reducing antioxidant power assay (FRAP) method was used based on the protocol of Bouayed et al. (41). In short, the FRAP reagent was prepared freshly by mixing acetate buffer (pH 3.6), 10 mM TPTZ solution in 40 mM HCl, and 20 mM iron(III) chloride solution (10:1:1, v/v/v). It was then warmed up (37°C in a water bath) before the application. Subsequently, 50 μl of the sample (appropriately diluted sample extracts or standard) was mixed with 1.5 ml of the FRAP reagent. After a 4-min incubation period, the absorbance was read at 593 nm vs. the blank. Quantification was carried out by external calibration curves using iron(II) sulfate solution (*n* = 7 concentrations between 100 and 2,000 μmol), and the antioxidant capacity was expressed as μmol Fe(II) per 100 g DW.

### 2.10. Statistical analysis

Normal distribution of data and equality of variance were determined through normality plots and box plots, respectively. When needed, data were log-transformed to fulfill the criteria of normal distribution. The effect of three factors on outcomes was studied. Multivariate models were then employed, with the concentration of trace elements, total phenolic content, total flavonoid content, as well as total antioxidant activity, as the observed (dependent) variables, variety (two levels), year (two levels), and treatment type (Se or Zn at two levels plus unamended control, i.e., five levels) as independent fixed factors. Biofortification levels were nested within biofortificant. Following significant Fisher's *F*-tests, all group-wise comparisons were carried out (Bonferroni *post hoc* tests). Following significant interactions, the models were further broken down to keep one of the interaction factors constant. A *P*-value of < 0.05 was regarded as statistically significant (two-sided). All analyses were performed using SPSS (version 19.0, IBM, Chicago, IL, USA).

Bivariate Pearson correlations were employed to study the relationship between total antioxidant activity (ABTS and FRAP) and trace elements, as well as total phenolics and total flavonoids, both for the Ambassador and Premium varieties.

Multivariable linear regression models (one for ABTS and one for FRAP) were created, following a backward elimination procedure starting with variety, total phenolics, total flavonoids, and all trace elements measured. For this purpose, *p*-values below 0.1 were considered for inclusion in the next step, while parameters with the highest *p*-values were step-wise excluded. This allowed for obtaining a model from the full set of variables (saturated model), where we automatically removed variables that did not contribute significantly to the *R*-square in the model.

## 3. Results

### 3.1. Overall effects

Combined analysis of variance showed that treatment (pooled years and varieties) significantly affected only total phenolic and total flavonoid contents in seeds. The growing year (pooled treatments and varieties) had a significant effect on all variables except for seed Mn concentration. Variety (pooled treatments and years) showed a significant effect on all variables, with the exception of total flavonoid content in seeds. In some cases, interactions were significant (Table 1).

**Table 1:** Combined ANOVA for the effects of year, variety, and treatment on Fe, Cu, and Mn concentrations, total phenolic content, total flavonoid content, and total antioxidant activity (ABTS and FRAP) of seeds.

	**DF**	**Fe mg/kg DW**	**Cu mg/kg DW**	**Mn mg/kg DW**	**TPC mg/100 g DW**	**TFC mg/100 g DW**	**ABTS mg/100 g DW**	**FRAP mmol/100 g DW**
Year (Y)	1	0.011	< 0.001	NS	< 0.001	< 0.001	< 0.001	0.001
Variety (V)	1	< 0.001	< 0.001	< 0.001	< 0.001	NS	< 0.001	< 0.001
Treatment (T)	4	NS	NS	NS	< 0.001	0.004	NS	NS
Y × V	1	NS	< 0.001	0.047	NS	NS	NS	< 0.001
Y × T	4	NS	0.023	0.002	0.005	0.016	NS	NS
V × T	4	NS	NS	< 0.001	0.001	NS	NS	NS
Y × V × T	4	NS	NS	0.009	NS	NS	NS	0.018

TPC, total phenolic content; TFC, total flavonoid content; DF, degrees of freedom; NS, not significant.

### 3.2. Concentration of trace elements in seeds

In 2014, treatment did not significantly impact the concentration of Fe vs. controls in both varieties. Ambassador had a significantly higher Fe concentration than Premium for all treatments except for Zn1. In 2015, treatment did not significantly influence the concentration of Fe vs. the control in Ambassador; however, both Se and Zn treatments significantly decreased the concentration of Fe vs. the control in Premium. Ambassador had a significantly higher Fe concentration than Premium for Se1 and Zn2. Growing year showed a significant effect on the concentration of Fe in Premium vs. Ambassador (Table 2).

**Table 2:** Effect of foliar application of Se and Zn, depending on variety and year, on seed Fe, Cu, and Mn concentrations.

**Year**	**Treatment**	**Fe (mg/kg DW)**		***p*-value**	**Cu (mg/kg DW)**		***p*-value**	**Mn (mg/kg DW)**		***p*-value**
		**Ambassador**	**Premium**		**Ambassador**	**Premium**		**Ambassador**	**Premium**	
2014	Control	55.0 ± 4.11^AB^	41.9 ± 3.19	**0.002**	6.90 ± 0.30	5.11 ± 0.21	**< 0.001**	8.85 ± 0.30	10.3 ± 1.27	0.076
	Se1	50.9 ± 4.94^A^	42.9 ± 3.23	**0.036**	6.37 ± 0.14	4.89 ± 0.27	**< 0.001**	9.51 ± 0.67	10.4 ± 0.50	0.072
	Se2	50.4 ± 3.21^A^	44.1 ± 2.94	**0.027**	6.74 ± 0.13	4.99 ± 0.13	**< 0.001**	9.51 ± 0.67	10.9 ± 0.68	**0.030**
	Zn1	50.4 ± 2.67^A^	47.8 ± 6.25	0.468	6.40 ± 0.23	5.33 ± 0.67	**0.023**	9.45 ± 0.46	11.6 ± 0.89	**0.005**
	Zn2	71.3 ± 17.4^B^	44.5 ± 2.66	**0.023**	6.41 ± 0.57	5.24 ± 0.27	**0.011**	9.79 ± 0.84	10.5 ± 0.48	0.190
	*p*-value	**0.015**	0.305		0.111	0.447		0.334	0.208	
2015	Control	55.4 ± 25.0	55.2 ± 3.56^C^	0.990	6.99 ± 0.63	5.10 ± 0.55	**0.004**	8.94 ± 0.68	12.0 ± 0.96^B^	**0.002**
	Se1	68.2 ± 2.79	50.3 ± 0.52^AB^	**< 0.001**	8.03 ± 0.90	5.31 ± 0.33	**0.001**	9.97 ± 0.61	9.92 ± 0.10^A^	0.877
	Se2	56.5 ± 24.3	51.4 ± 0.93^B^	0.691	8.45 ± 0.59	5.44 ± 0.15	**< 0.001**	10.0 ± 0.56	9.99 ± 0.18^A^	0.864
	Zn1	55.7 ± 9.75	51.3 ± 0.54^B^	0.398	8.29 ± 0.77	5.39 ± 0.06	**< 0.001**	8.97 ± 1.16	9.80 ± 0.08^A^	0.200
	Zn2	63.6 ± 3.01	46.9 ± 0.78^A^	**< 0.001**	8.39 ± 1.15	5.29 ± 0.15	**0.002**	10.3 ± 0.62	9.94 ± 0.09^A^	0.273
	*p*-value	0.738	**< 0.001**		0.135	0.576		0.057	**< 0.001**	
	*p-value across	0.306	**< 0.001**		**< 0.001**	0.078		0.318	0.071	

Control, without Se/Zn; Se1, 50 g Se/ha; Se2, 100 g Se/ha; Zn1, 375 g Zn/ha; Zn2, 750 g Zn/ha, mean ± SD; n = 4. Means within a column followed by non-identical letters are significantly different. *P*-values in the same row mean the effect of Se/Zn dose. *P*-values in the same column mean the effect of variety. ^*^P-values across refer to the effect of year. *P*-values in bold are statistically significant.

For both 2014 and 2015, treatment did not significantly affect Cu concentration vs. controls in both varieties. Ambassador had significantly higher Cu concentration vs. Premium for all treatments in both years. Growing year had a significant influence on Cu concentration in Ambassador compared with Premium (Table 2).

In 2014, treatment did not significantly impact the concentration of Mn compared with controls in both varieties. Premium showed slightly but significantly higher Mn concentration than Ambassador for Se2 and Zn1. In 2015, treatment failed to show (*p* = 0.057) a significant influence on the concentration of Mn compared with the control in Ambassador. Both Se and Zn applications significantly reduced the concentration of Mn vs. the control in Premium. Premium exhibited significantly higher Mn concentration than Ambassador for the control. Growing year had no significant impact on the concentration of Mn in both varieties (Table 2).

### 3.3. Total phenolic content (TPC) in seeds

**Figure 1:**
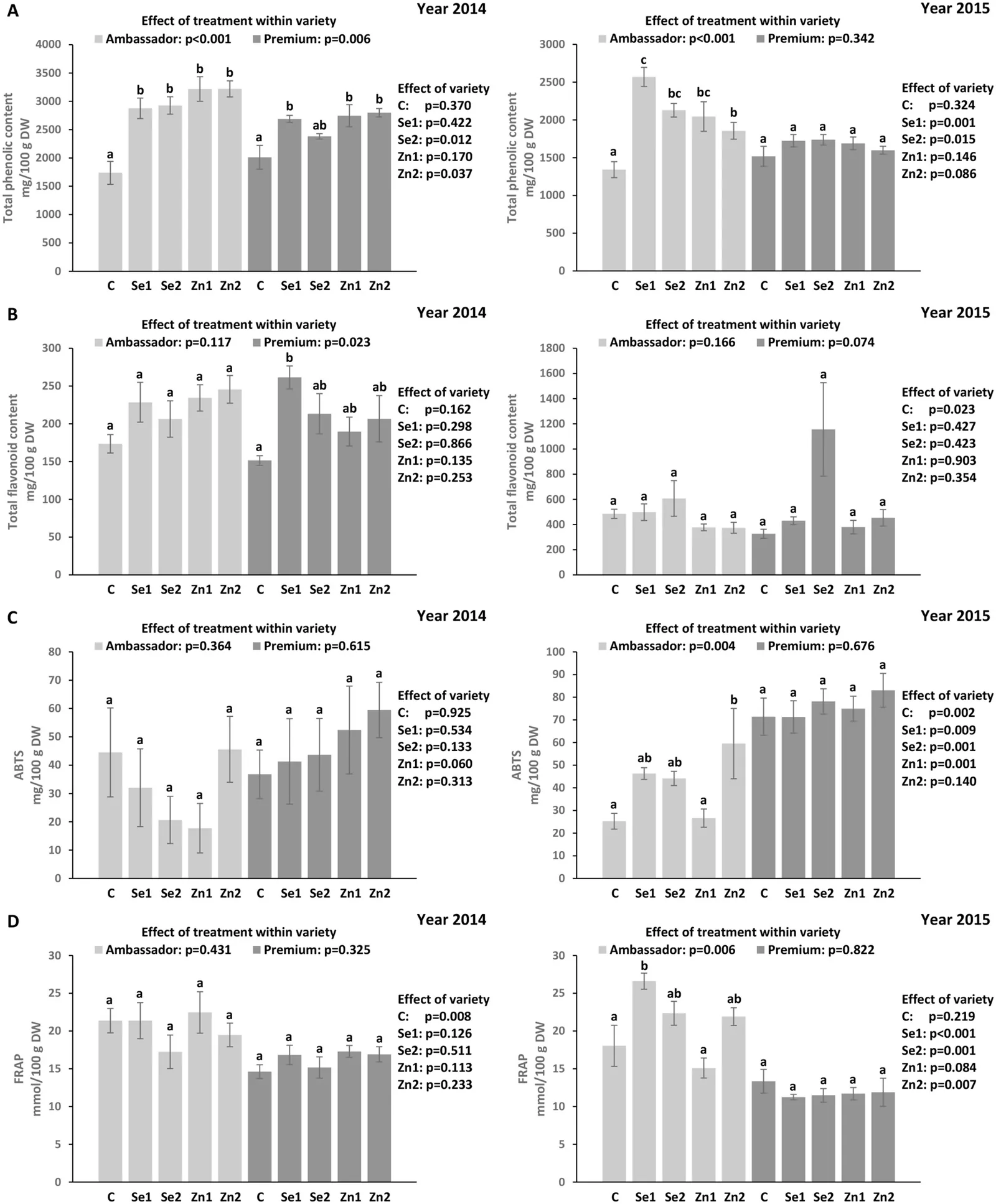
Effect of foliar application of Se and Zn in two growing seasons (2014 and 2015) on total phenolic content **(A)**, total flavonoid content **(B)**, and total antioxidant activity (ABTS and FRAP) [**(C, D)**, respectively] of pea seeds. C (control), without Se and Zn; Se1, 50 g Se/ha; Se2, 100 g Se/ha; Zn1, 375 g Zn/ha; Zn2, 750 g Zn/ha; mean ± SD; *n* = 4. Bars not sharing the same superscript are significantly different within varieties. *P*-values on the right side of the figure show the effect of treatment across the two varieties (i.e., Ambassador vs. Premium).

In 2014, Zn2 followed by Zn1, Se2, and Se1 significantly increased TPC compared with the control in Ambassador, while Zn2 following Zn1 and Se1 significantly increased TPC vs. the control in Premium. Ambassador had significantly higher TPC than Premium for Se2 and Zn2. In 2015, treatment did not significantly affect TPC vs. the control in Premium, while Se1 followed by Se2, Zn1, and Zn2 significantly increased TPC vs. the control in Ambassador. Ambassador also exhibited significantly higher TPC than Premium for Se1 and Se2 (Figure 1A). Growing year showed a significant effect on TPC in both varieties (*p* < 0.001).

### 3.4. Total flavonoid content (TFC) in seeds

In 2014, treatment did not significantly affect TFC vs. the control in Ambassador, while the Se1 treatment significantly increased TFC vs. the control in Premium. No significant differences were found for TFC between Ambassador and Premium for all treatments. In 2015, treatment did not significantly influence TFC vs. controls in both varieties. Ambassador showed significantly higher TFC than Premium for the control (Figure 1B). Growing year had a significant effect on TFC in both varieties (*p* < 0.001).

### 3.5. ABTS of seeds

In 2014, treatment did not significantly impact ABTS compared with controls of both varieties. No significant differences were observed for ABTS between Ambassador and Premium for all treatments. In 2015, treatment did not significantly affect ABTS vs. the control of Premium, while the Zn2 treatment significantly increased ABTS vs. the control of Ambassador. Premium showed significantly higher ABTS than Ambassador for all treatments with the exception of Zn2 (Figure 1C). Growing year had a significant effect on ABTS of Ambassador (*p* = 0.029) and Premium (*p* < 0.001).

### 3.6. FRAP of seeds

In 2014, treatment did not significantly influence FRAP vs. controls of both varieties. Ambassador had significantly higher FRAP than Premium for controls. In 2015, treatment did not significantly impact FRAP vs. the control of Premium, while the Se1 treatment significantly increased FRAP vs. the control of Ambassador. The latter showed significantly elevated FRAP vs. Premium for Se1, Se2, and Zn2 (Figure 1D). Growing year showed a significant influence on FRAP of Premium (*p* < 0.001) compared with Ambassador.

### 3.7. Correlations

**Figure 2:**
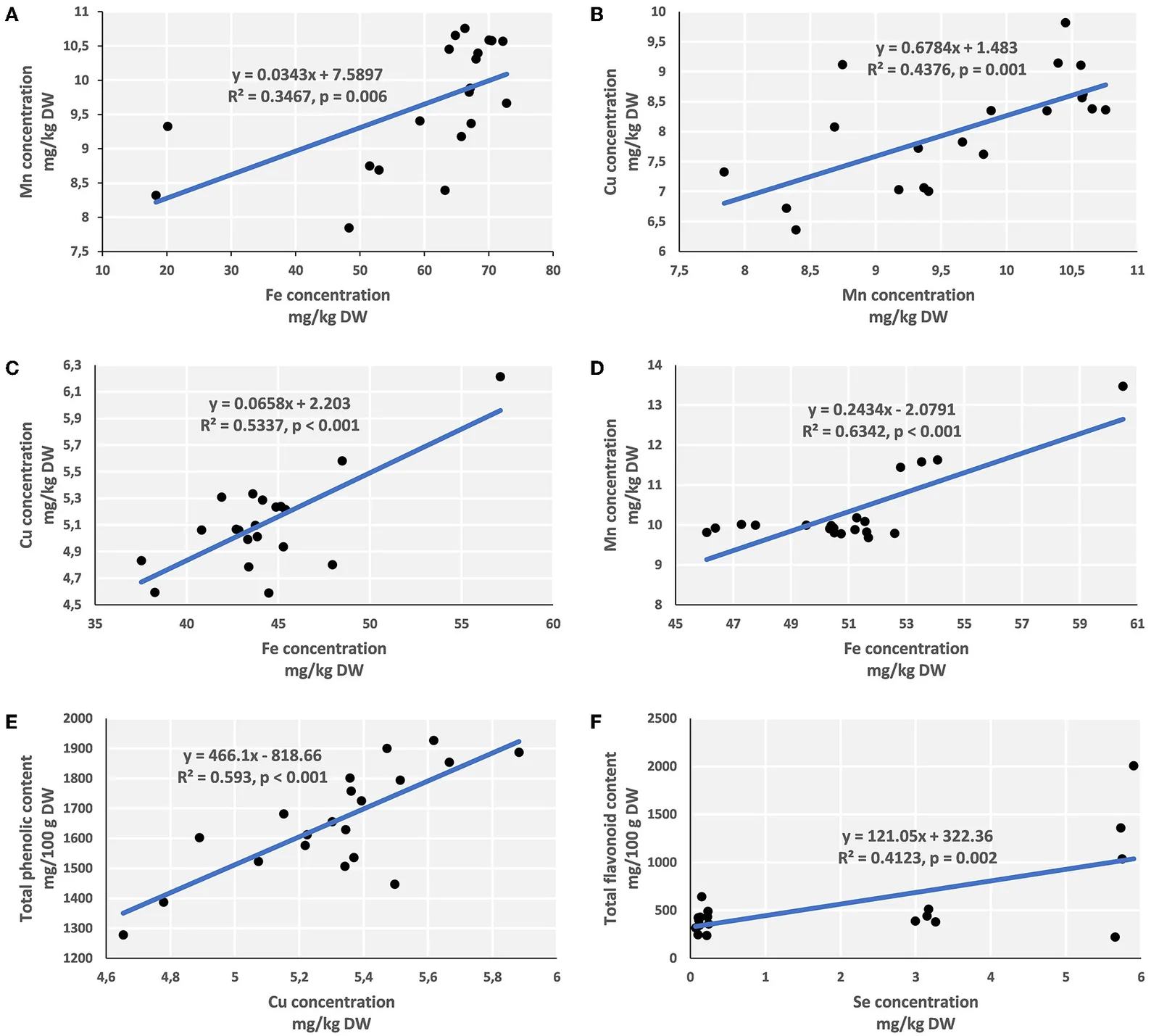
Bivariate correlations between Mn and Fe concentrations for Ambassador from the 2015 growing season **(A)**, between Cu and Mn concentrations for Ambassador from the 2015 growing season **(B)**, between Cu and Fe concentrations for Premium from the 2014 growing season **(C)**, between Mn and Fe concentrations for Premium from the 2015 growing season **(D)**, between total phenolics and Cu concentration for Premium from the 2015 growing season **(E)**, and between total flavonoids and Se concentration for Premium from the 2015 growing season **(F)**.

Overall, the strongest positive correlations were generally found between Mn and Fe concentrations, for both years and varieties, though not always reaching statistical significance. Furthermore, Zn concentration and ABTS always correlated positively, though not always significantly. In addition, significant positive correlations were observed between total phenolics and Cu concentration, and between total flavonoids and Se concentration for Premium from the 2015 growing season, between Fe and Cu concentrations for Premium from the 2014 growing season, and between Mn and Cu and Fe concentrations for Ambassador from the 2015 growing season (Figure 2). The full matrix of correlations is shown in Supplementary Table S2.

For Ambassador, Cu concentration was significantly and negatively correlated with seed dry matter (*r*^2^ = 0.1998), number of seeds/pod (*r*^2^ = 0.1927), pod length (*r*^2^ = 0.1544), and pod perimeter (*r*^2^ = 0.3091). A significant negative correlation was also observed between total flavonoids and seed dry matter (*r*^2^ = 0.2079), number of seeds/pod (*r*^2^ = 0.1232), pod length (*r*^2^ = 0.1109), and pod perimeter (*r*^2^ = 0.3919). Total phenolics showed a significant and positive correlation with the number of seeds/pod (*r*^2^ = 0.3158), pod length (*r*^2^ = 0.0980), and pod perimeter (*r*^2^ = 0.3709). For Premium, seed dry matter was significantly negatively correlated with Fe concentration (*r*^2^ = 0.1274) and ABTS (*r*^2^ = 0.3003). Pod length showed a significant and negative correlation with FRAP (*r*^2^ = 0.1436). Pod perimeter was significantly positively correlated with total phenolics (*r*^2^ = 0.4692) and FRAP (*r*^2^ = 0.3434), while it was significantly negatively correlated with Fe concentration (*r*^2^ = 0.4122), Cu concentration (*r*^2^ = 0.1253), total flavonoids (*r*^2^ = 0.1459), and ABTS (*r*^2^ = 0.4032) (Supplementary Table S3).

### 3.8. Regression analyses

The variety of pea seeds did not result in a statistically significant effect in both models (ABTS and FRAP), thus combined results were analyzed. For ABTS, the final model included a constant, Zn, Mn, Cu, and total phenolics, with a regression coefficient R of 0.577 (Table 3). For FRAP, the final model included a constant, Se, Cu, and total phenolics, with a regression coefficient R of 0.696 (Table 4).

**Table 3:** Regression model for the association between trace elements, total phenolics and ABTS based on multivariable linear regression modeling.

**Variables**	**Beta-non standar- dized**	**Standard error of beta**	**Beta-standar-dized**	***p*-value**
Constant	−12.574	34.066		0.713
Cu	−7.471	2.935	−0.369	**0.013**
TPC	−0.009	0.005	−0.213	0.072
Zn	0.828	0.407	0.316	**0.046**
Mn	9.406	2.617	0.355	**0.001**

Overall R was 0.577. *P*-values in bold indicate a statistically significant difference.

TPC, total phenolic content.

**Table 4:** Regression model for the association between trace elements, total phenolics and FRAP based on multivariable linear regression modeling.

**Variables**	**Beta-non standar- dized**	**Standard error of beta**	**Beta-standar-dized**	***p*-value**
Constant	−2.1306	−2.5285		0.402
Se	−0.0003	−0.0002	−0.150	0.080
Cu	2.1018	0.3316	0.531	**< 0.001**
TPC	0.0031	0.0007	0.379	**< 0.001**

Overall R was 0.696. *P*-values in bold indicate a statistically significant difference.

TPC, total phenolic content.

## 4. Discussion

In the present study, we examined the influence of the foliar application of selenate and zinc oxide at the flowering stage on two pea varieties. The concentration of selected trace elements, total phenolic content, total flavonoid content, as well as the total antioxidant activity in seeds, were determined. Though treatments did not improve concentrations of trace elements, they generally enhanced total phenolic content and in part (though not consistently) total flavonoid content and total antioxidant activity (Tables 1, 2, Figure 1).

Pea was selected as it represents an important legume and nutritious staple with global importance for food security. The varieties, Ambassador and Premium, were chosen, on the one hand, due to their high-yielding performance, and on the other hand, because studies have suggested that dark-seeded varieties of pea seeds have a high potential to accumulate phenolics (43–46). Selenium and Zn solutions were administered *via* foliar application, as this approach reduced the effect of soil properties on interactions between the examined minerals (47). Previous findings employing foliar Se and Zn fertilization suggested that field peas, especially due to their higher protein concentration, may show a larger potential for Se and Zn uptake and therefore seed accumulation than cereals (48, 49). Selenium as selenate was applied due to its recognized high efficacy for foliar uptake (50). Although the essentiality of Se to higher plants is still controversial, beneficial effects of Se applications at low concentrations have been demonstrated, including for plant growth, development and yield, as well as enhanced resistance to abiotic stresses (51). Zinc oxide was employed following recommendations by the agro-industry. In contrast to Se, Zn is an essential trace element needed for plant growth and quality (52). Concentrations of trace elements and bioactive compounds were examined, as these are also needed for plant growth and/or their intakes have been related to human health outcomes.

Our previous study highlighted that foliar-applied sodium selenate and zinc oxide had minimal overall effects on the growth parameters of pea (39). This study further showed that selenate improved seed Se accumulation in both employed varieties in a dose-dependent fashion. Premium accumulated greater amounts of Se in seeds than Ambassador. The most pronounced Se accumulation was found in seeds of Premium treated with 100 g Se/ha (7.84 mg/kg DW) vs. the control (0.16 mg/kg). Contrarily, zinc oxide did not significantly affect seed Zn accumulation (Supplementary Table S1).

The present study revealed that selenate and zinc oxide did not improve concentrations of Fe, Cu, and Mn in pea seeds (Tables 1, 2), suggesting that Se and Zn did not impair the transport of the other trace elements. In theory, at low concentrations, Se may act as an antioxidant in plants, reducing lipid peroxidation, whereas at higher levels, it could act as a pro-oxidant, increasing the accumulation of lipid peroxidation products (53–55). Feng et al. (56) indicated that there can be associations between Se, Fe, and lipid peroxidation in plants, and suggested that the ambivalent effects of Se on plants may occur through the downregulation of Fe at low Se doses and the upregulation of Fe at high Se doses, as Fe may constitute a pro-oxidant following the Haber–Weiss reaction (57).

However, similar to the present findings, Poblaciones and Rengel demonstrated that foliar-applied selenate and zinc sulfate individually and in several combinations (at early seed filling) did not significantly affect Fe concentration in pea seeds (48, 49). Similarly, Kayan et al. (58) found that Fe concentration in seeds of chickpea was slightly, though not significantly, increased following foliar-applied Zn (zinc chelate and zinc sulfate) vs. controls. In contrast, another study by Poblaciones and Rengel (59) that examined the effect of all combinations of soil/foliar applications (before flowering and at the early seed-filling stage) of zinc sulfate on field pea, showed that seed Fe concentrations were significantly reduced (vs. the control) by soil Zn application. It could be argued that this may have been due to competitive reasons, as Fe and Zn can compete for similar absorption pathways in the plant root, as reviewed previously (60, 61). The uptake of trace elements by plants is affected by multiple transporters, many of which may transport more than one. For example, the Fe-regulated transporter (IRT), expressed in roots, is induced mainly by Fe deficiency (also by Zn application during Fe deficiency), and can transport Fe, Mn, Cu, Zn, and probably other divalent cations (62–64). Sharing similar uptake/transport systems by these ions may cause competition among them, resulting in antagonism and reduced accumulation (64). It is also worth noting that grain Zn and Fe concentrations may correlate significantly and positively with grain protein concentration (65, 66), which is in line with our recent study on pea seeds (47), indicating that genes influencing the accumulations of Zn, Fe, and protein synthesis are closely linked (67).

Our study showed that selenate enhanced total phenolic content and in part (though not consistently) total flavonoid content in pea seeds (Table 1, Figures 1A, B). Previous research also demonstrated that foliar-applied selenate positively affected total phenolic content in pea seeds (68), curly endive (69), and cauliflower florets (70), as well as total flavonoid content in Indian mustard leaves (71) and tomato fruits (72). The present study further revealed that selenate improved in part (though not consistently) the total antioxidant activity (FRAP) of pea seeds (Table 1, Figures 1C, D). Beneficial effects of foliar-applied selenate on total antioxidant activity were also reported previously for pea seeds (68) and cauliflower florets (70), although different tests were employed (2,2-diphenyl-1-picrylhydrazyl; DPPH and/or photochemiluminescence).

D'Amato et al. (73) emphasized that stress in the plant, induced by increased Se levels, activates the phenylpropanoid pathway involved in the biosynthesis of phenolic compounds (including phenolic acids), which may enhance the scavenging capacity of reactive oxygen species (ROS). The authors also indicated that these effects depend on plant species and plant growth stage, as well as Se treatments and forms. Our study confirmed not only the role of Se treatments but also of variety on the accumulation of total phenolics in pea seeds (Figure 1A). Groth et al. (74), in their study on apples, highlighted an inverse correlation between polyphenol oxidase (PPO) activity and total phenolic content, as the enzyme catalyzes the oxidation of phenolic compounds, which could explain the effect of Se biofortification, as well as the variety-specific differences. It is also worth stressing that Zhu et al. (72) indicated that proteins involved in the phenylpropanoid biosynthetic pathway, such as NAD-dependent epimerase (P230), oxidoreductase zinc-binding dehydrogenase (P242), and acyl-CoA synthetase (P252), were upregulated in Se-enriched tomato fruit, resulting in an increased total flavonoid content.

Our study further demonstrated that zinc oxide did not improve total flavonoid content, while it enhanced total phenolic content and in part (though not consistently) total antioxidant activity (ABTS) of pea seeds (Table 1, Figure 1). The fact that zinc oxide did not improve the accumulation of Zn, while it improved the accumulation of phenolics in seeds (Supplementary Table S1, Figure 1A) may suggest that exposure of pea plants to Zn was a sufficient stimulus to induce phenolic biosynthesis pathways. It has been revealed that Zn application is associated with the increased activity of enzymes of secondary plant metabolite pathways, namely shikimate dehydrogenase, phenylalanine ammonialyase (PAL), and PPO (75). Song et al. (76) showed, though on Zn-deficient soil, that foliar-applied zinc sulfate improves the accumulation of total phenols, flavonoids, flavanols, tannins, and anthocyanins in grape berries. DFR and LDOX genes, key genes for polyphenol formation, can catalyze the production of proanthocyanidins and anthocyanidins (77, 78). In the study by Song et al. (76), zinc sulfate significantly affected the expression of genes involved in phenolic (especially flavonol and anthocyanin) biosynthetic pathways (*VvPAL, VvSTS29, VvCHS, VvCHI, VvF3H, VvFLS4, VvDFR, VvLDOX*, and *VvMYBF1*) throughout berry development, fostering phenolic accumulation. The authors also pointed out that sucrose is a positive regulator of the biosynthesis of phenolics, particularly flavonoids (79), and the beneficial effect of Zn application on photosynthesis and sugar accumulation may improve the biosynthesis of flavonoids.

Finally, the present study showed significant relationships, among others, between Mn, Cu, and Fe concentrations, as well as between Fe and Cu concentrations in pea seeds (Figures 2A–D, Supplementary Table S2). The association between micro-minerals in plants may be affected by a number of factors that include species of the plant as well as genotype, dose and form of fertilizer, route of fertilizer application, cultivation aspects, characteristics of the soil, and antagonism and/or synergism between various elements, as emphasized by Malka et al. (47). Imbalance of trace minerals such as Fe, Cu, Mn, Zn, and Se, which are cofactors for antioxidant enzymes (51, 80), may affect the presence of bioactive compounds and total antioxidant activity of plants. Indeed, our research revealed significant positive relationships between total phenolics and Cu concentration (Figure 2E), as well as between total flavonoids and Se concentration (Figure 2F) for Premium seeds. Based on the linear multivariable regression models, Zn, Mn, Cu, and total phenolics were the best explanatory parameters for ABTS (Table 3), while Se, Cu, and total phenolics were the best explanatory variables for FRAP (Table 4), demonstrating the general importance of these trace elements and total phenolics for the total antioxidant activity of pea seeds. Previous studies on apples (81) and baby mustard (82) also showed that total phenolics were associated with total antioxidant activity (ABTS and FRAP). Furthermore, our previous study revealed that other compounds, including Ca, Mg, K, Na, soluble solids, protein, chlorophyll a and b, total chlorophyll, total carotenoids, and total condensed tannins, did not contribute substantially to the total antioxidant activity (ABTS and FRAP) of pea seeds (47).

## 5. Conclusion

Taken together, our study highlighted that selenate and zinc oxide, foliar applied at the flowering stage, generally enhanced total phenolic content in pea seeds. In addition, in part (though not consistently), selenate improved total flavonoid content and FRAP, while zinc oxide increased ABTS of seeds. Furthermore, the importance of trace elements for total phenolics and antioxidant activity was emphasized by multivariable regression models. Additional investigations are required to reveal molecular mechanisms regulating the accumulation of phenolic compounds in pea upon foliar-applied Se/Zn. Furthermore, although growing season is expected to play a role, the evaluation of the importance of climate conditions on the investigated parameters requires additional experiments under field conditions, which was not the case in the present study. This research is important in terms of producing pea/pea products rich in secondary plant metabolites with additional human health benefits. We conclude that foliar biofortification with trace elements may have the potential to boost phytochemical antioxidants in crops.

## Data availability statement

The original contributions presented in the study are included in the article/Supplementary material, further inquiries can be directed to the corresponding author.

## Author contributions

MM designed and carried out the experiments, as well as prepared the first version of the manuscript. TB was involved in the analysis, statistical interpretation, and manuscript writing. GK was involved in performing the analysis. GD was involved in the conceptualization, supervision of the project, and writing of the manuscript. AH organized, designed, and supervised the experiments in Nitra. All authors have read and agreed to the published version of the manuscript.
