# Correction: Enhanced accumulation of phenolics in pea (*Pisum sativum* L.) seeds upon foliar application of selenate or zinc oxide

**DOI:** 10.3389/fnut.2026.1893819

**Published:** 2026-07-10

**Authors:** Maksymilian Malka, Gijs Du Laing, Gabriela Kurešová, Alžbeta Hegedüsová, Torsten Bohn

**Affiliations:** 1 Laboratory of Analytical Chemistry and Applied Ecochemistry, Department of Green Chemistry and Technology, Faculty of Bioscience Engineering, Ghent University, Ghent, Belgium; 2 Department of Plant Protection, Crop Research Institute, Prague, Czechia; 3 Institute of Horticulture, Faculty of Horticulture and Landscape Engineering, Slovak University of Agriculture in Nitra, Nitra, Slovakia; 4 Nutrition and Health Research Group, Department of Precision Health, Luxembourg Institute of Health, Strassen, Luxembourg

**Keywords:** oxidative stress, micronutrients, trace elements, polyphenols, legume biofortification, food security, mineral deficiency, foliar Se/Zn application

In the Abstract, the following sentence was erroneously written as, “In addition, 50 g of Se/ha increased TFC vs. the control (261 vs. 151 mg/100 g DW) in Premium (from 2014), 750 g of Zn/ha increased ABTS vs. the control (25.2 vs. 59.5 mg/100 g DW) in Ambassador (from 2015), and 50 g of Se/ha increased FRAP vs. the control (26.6 vs. 18.0 mmol/100 g DW) in Ambassador (from 2015).” The correct sentence should read as, “In addition, 50 g of Se/ha increased TFC vs. the control (261 vs. 151 mg/100 g DW) in Premium (from 2014), 750 g of Zn/ha increased ABTS vs. the control (59.5 vs. 25.2 mg/100 g DW) in Ambassador (from 2015), and 50 g of Se/ha increased FRAP vs. the control (26.6 vs. 18.0 mmol/100 g DW) in Ambassador (from 2015).”

The original version of this article has been updated.

