# Supplementary material for: Enhanced accumulation of phenolics in pea (Pisum sativum L.) seeds upon foliar application of selenate or zinc oxide
Source: Front Nutr. 2023 Mar 30;10:1083253. doi: 10.3389/fnut.2023.1083253 (PMC10097936; doi:10.3389/fnut.2023.1083253)
Supplement: Supplementary file 1 [file Data_Sheet_1.docx]

**Supplementary material**

**Table S1:** Effect of foliar-applied Se and Zn, variety and year on seed Se and Zn concentrations.

|  |  | **Se (mg/kg DW)** | |  | **Zn (mg/kg DW)** | |  |
| --- | --- | --- | --- | --- | --- | --- | --- |
| **Year** | **Treatment** | **Ambassador** | **Premium** | **p-value** | **Ambassador** | **Premium** | **p-value** |
| 2014 | Control | 0.08 ± 0.01^A^ | 0.16 ± 0.06^A^ | **0.039** | 41.6 ± 3.47 | 25.8 ± 3.15 | **0.001** |
|  | Se1 | 2.59 ± 0.45^B^ | 4.28 ± 1.69^B^ | 0.180 | 34.4 ± 1.64 | 25.2 ± 2.87 | **0.002** |
|  | Se2 | 4.87 ± 3.20^B^ | 7.84 ± 2.97^B^ | 0.323 | 38.2 ± 6.15 | 26.9 ± 1.77 | **0.008** |
|  | Zn1 | 0.09 ± 0.01^A^ | 0.18 ± 0.06^A^ | **0.013** | 38.5 ± 2.45 | 30.1 ± 5.36 | **0.027** |
|  | Zn2 | 0.08 ± 0.01^A^ | 0.18 ± 0.07^A^ | **0.031** | 35.4 ± 1.25 | 29.4 ± 3.46 | **0.018** |
|  | p-value | **<0.001** | **<0.001** |  | 0.076 | 0.216 |  |
| 2015 | Control | 0.12 ± 0.02^A^ | 0.09 ± 0.02^A^ | **0.033** | 55.3 ± 1.97^A^ | 44.9 ± 2.18^BC^ | **<0.001** |
|  | Se1 | 1.26 ± 0.04^CD^ | 3.14 ± 0.11^D^ | **<0.001** | 55.7 ± 1.62^A^ | 41.3 ± 0.42^A^ | **<0.001** |
|  | Se2 | 2.99 ± 0.08^D^ | 5.76 ± 0.10^E^ | **<0.001** | 54.9 ± 1.52^A^ | 42.8 ± 0.74^AB^ | **<0.001** |
|  | Zn1 | 0.90 ± 1.33^BC^ | 0.23 ± 0.01^C^ | 0.338 | 45.0 ± 7.49^B^ | 44.2 ± 0.28^BC^ | 0.918 |
|  | Zn2 | 0.15 ± 0.01^AB^ | 0.13 ± 0.02^B^ | **<0.001** | 52.0 ± 1.15^AB^ | 45.3 ± 1.10^C^ | 0.051 |
|  | p-value | **<0.001** | **<0.001** |  | **0.003** | **<0.001** |  |
|  | ^*^p-value across | 0.074 | 0.097 |  | **<0.001** | **<0.001** |  |

Control: without Se / Zn; Se1: 50 g Se/ha; Se2: 100 g Se/ha; Zn1: 375 g Zn/ha; Zn2: 750 g Zn/ha; mean ± SD; n = 4. Means within a column followed by different letters are significantly different. P-values in the same row mean the effect of Se / Zn dose. P-values in the same column mean the effect of variety. ^*^P-values across refer to the effect of year. P-values in bold are statistically significant. Results adapted from Malka et al. (39).

**Table S2:** Pearson correlation coefficients between trace elements, total phenolics, total flavonoids and total antioxidant activity (ABTS and FRAP) evaluated for seeds of two pea varieties (Ambassador and Premium) grown in two seasons (2014 and 2015). In light grey – 2014 growing season, in dark grey – 2015 growing season.

| **Ambassador variety** | | | | | | | | | |
| --- | --- | --- | --- | --- | --- | --- | --- | --- | --- |
|  | Se | Zn | Fe | Cu | Mn | TPC | TFC | FRAP | ABTS |
| Se |  | 0.397 | 0.056 | 0.300 | 0.397 | 0.507* | 0.384 | 0.030 | 0.268 |
| Zn | -0.361 |  | 0.156 | -0.038 | 0.526* | 0.163 | 0.310 | 0.178 | 0.491* |
| Fe | -0.332 | -0.126 |  | 0.341 | 0.589** | 0.264 | -0.473* | 0.169 | 0.369 |
| Cu | 0.021 | 0.406 | -0.118 |  | 0.662** | 0.579** | -0.174 | 0.272 | 0.261 |
| Mn | -0.083 | -0.020 | 0.485^*^ | -0.011 |  | 0.475* | -0.098 | 0.525* | 0.555* |
| TPC | 0.193 | -0.269 | 0.064 | -0.308 | 0.413 |  | 0.160 | 0.293 | 0.488* |
| TFC | -0.281 | -0.184 | -0.038 | -0.095 | 0.269 | 0.415 |  | 0.079 | 0.146 |
| FRAP | -0.308 | 0.258 | 0.206 | -0.159 | 0.217 | -0.251 | -0.132 |  | 0.483* |
| ABTS | -0.329 | 0.323 | -0.178 | 0.154 | 0.078 | -0.028 | 0.060 | 0.255 |  |
| **Premium variety** | | | | | | | | | |
|  | Se | Zn | Fe | Cu | Mn | TPC | TFC | FRAP | ABTS |
| Se |  | -0.609** | 0.002 | 0.249 | -0.311 | 0.352 | 0.642** | 0.002 | -0.179 |
| Zn | -0.356 |  | 0.184 | 0.152 | 0.469* | -0.044 | -0.139 | 0.197 | 0.039 |
| Fe | 0.034 | 0.450^*^ |  | 0.117 | 0.796** | 0.046 | 0.003 | -0.061 | 0.263 |
| Cu | -0.287 | 0.513^*^ | 0.731^**^ |  | -0.026 | 0.770** | 0.158 | 0.550* | 0.147 |
| Mn | 0.136 | -0.341 | 0.308 | -0.005 |  | -0.141 | -0.198 | 0.029 | 0.285 |
| TPC | -0.057 | 0.412 | 0.343 | 0.277 | 0.097 |  | -0.020 | 0.372 | -0.145 |
| TFC | 0.315 | 0.197 | 0.201 | 0.160 | -0.202 | 0.450^*^ |  | -0.085 | 0.109 |
| FRAP | 0.043 | -0.321 | 0.009 | 0.161 | 0.303 | 0.183 | 0.057 |  | 0.340 |
| ABTS | -0.318 | 0.577^**^ | 0.105 | 0.113 | -0.255 | 0.462^*^ | 0.384 | -0.256 |  |

TPC: total phenolic content; TFC: total flavonoid content. Level of significance: *p<0.05, **p<0.01.

**Table S3:** Pearson correlation coefficients between growth parameters and trace elements, total phenolics, total flavonoids and total antioxidant activity (ABTS and FRAP) evaluated for seeds of two pea varieties (Ambassador and Premium).

| **Variety /**  **growth parameter** | **Fe** | **Cu** | **Mn** | **TPC** | **TFC** | **ABTS** | **FRAP** |
| --- | --- | --- | --- | --- | --- | --- | --- |
| **Ambassador** |  |  |  |  |  |  |  |
| Seed dry matter | -0.015 | -0.447^**^ | -0.024 | 0.182 | -0.456^**^ | 0.201 | 0.083 |
| Number of seeds/pod | 0.063 | -0.439^**^ | 0.095 | 0.562^**^ | -0.351^*^ | -0.125 | 0.115 |
| Pod length | -0.263 | -0.393^*^ | -0.087 | 0.313^*^ | -0.333^*^ | -0.189 | -0.152 |
| Pod perimeter | -0.143 | -0.556^**^ | 0.014 | 0.609^**^ | -0.626^**^ | -0.056 | -0.039 |
| **Premium** |  |  |  |  |  |  |  |
| Seed dry matter | -0.357^*^ | -0.268 | 0.126 | 0.247 | -0.124 | -0.548^**^ | 0.280 |
| Number of seeds/pod | -0.230 | -0.060 | -0.057 | -0.051 | -0.142 | -0.272 | 0.013 |
| Pod length | 0.163 | 0.119 | 0.097 | -0.254 | 0.199 | 0.115 | -0.379^*^ |
| Pod perimeter | -0.642^**^ | -0.354^*^ | 0.202 | 0.685^**^ | -0.382^*^ | -0.635^**^ | 0.586^**^ |

TPC: total phenolic content; TFC: total flavonoid content. Level of significance: ^*^p<0.05, ^**^p<0.01.
